# Supplementary material for: What Are the Most Effective Factors in Determining Future Exacerbations, Morbidity Weight, and Mortality in Patients with COPD Attack?
Source: Medicina (Kaunas). 2022 Jan 21;58(2):163. doi: 10.3390/medicina58020163 (PMC8880362; doi:10.3390/medicina58020163)
Supplement: Supplementary file 1 [file medicina-58-00163-s001.zip › medicina-1480473-supplementary.pdf]

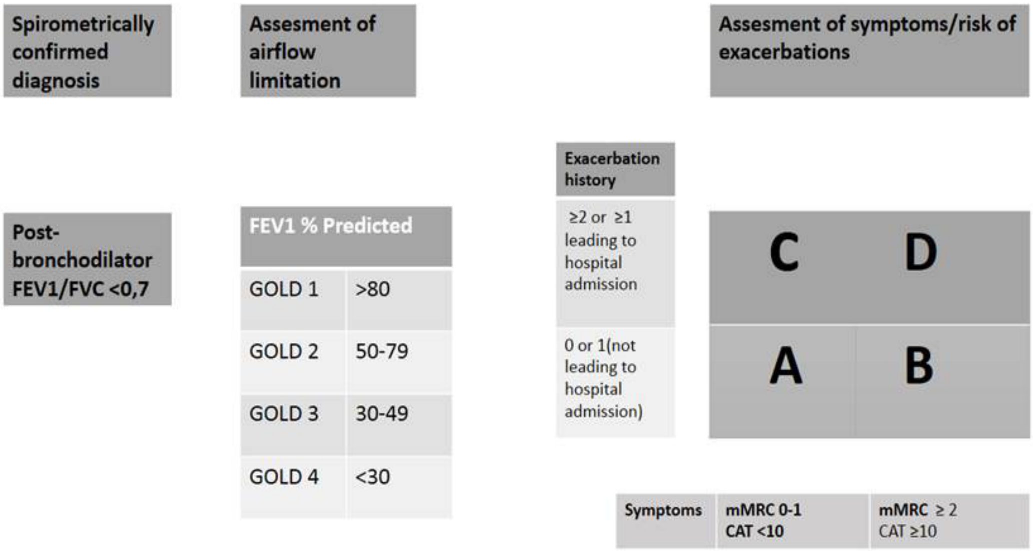

Supplementary Figure S1- The refined ABCD assessment tool according to Gold 2021 (FEV1: Forced expiratory volume in first second , FVC: Forced vital capacity , mMRC: Modified Medical Research Council , CAT : COPD Assessment Test).

Supplementary Table S1-COPD Assessment Test (CAT)

**CAT**		
**I never cough**	1 2 3 4 5	I cough all the time
**I have no phlegm (mucus) in my chest at all**	1 2 3 4 5	My chest is completely full of phlegm
**My chest does not feel tight at all**	1 2 3 4 5	My chest feels very tight
**When I walk up ahill or one flight of stairs I am not breathless**	1 2 3 4 5	When I walk up ahill or one flight of stairs I am very breathless
**I am not limited doing any activities at home**	1 2 3 4 5	I am very limited doing activities at home
**I am confident leaving my home despite my lung condition**	1 2 3 4 5	I am not at all confident leaving my home because of my lung condition
**I sleep soundly**	1 2 3 4 5	I don’t sleep soundly because of my lung condition
**I have lots of energy**	1 2 3 4 5	I have no energy at all

Supplementary Table S2- mMRC Dyspnea Scale

**mMRC**	
**Grade 0**	Not troubled by breathlessness except on strenuous exercise
**Grade 1**	Short of breath ehrn hurrying or walking up a slight hill
**Grade 2**	Walk slower than contemporaries on the level because of breathlessness or has to stop for breath walking at own pace
**Grade 3**	Stops for breath after walking 100 m or after a few minutes onthe level
**Grade 4**	Too breathless to leave the house or breathless when dressing or undressing

Supplementary Table S3- Borg Dyspnea Scale

SCORE	DYSPNEA
0	None
0,5	Very, very light
1	Very light
2	Light
3	Moderate
4	A little intense
5	Intense
6	
7	Very intense
8	
9	Very, very intense
10	Maximum

Supplementary Table S4-Charlson Comorbidity Index

COMORBIDITY	SCORE
**Miyocardial Infarction**	1
**Congestive Heart Failure**	1
**Peripheral Vascular Disease**	1
**Cerebrovascular Disease**	1
**Dementia**	1
**Chronic Pulmonary Disease**	1
**Rheumatologic Disease**	1
**Peptic Ulcer Disease**	1
**Mild Liver Disease**	1
**Diabetes**	1
**Hemiplegia**	2
**Moderate-to-severe Renal Disease**	2
**Diabetes With Chronic Cpmğlications**	2
**Cancer Without Metastases**	2
**Leukemia**	2
**Lymphoma**	2
**Moderate-to-severe Lung Disease**	3
**Metastatic Solid Tumor**	6
**Acquired Immune Deficiency Syndrome**	6

Supplementary Table S5A- General data of the patients

		Min-Max			Median	Mean ±s.d./n-%		
Age		40,0	-	91,0	66,5	66,4	±	10,7
Gender	Female					21		13,1%
	Male					139		86,9%
BMI		15,1	-	41,6	24,8	25,7	±	5,0
COPD duration (years)		0,0	-	34,0	6,0	7,9	±	7,0
Active smoker						50		54,4%
Non-smoker						23		14,4%
Ex-smoker						87		31,2%
BORG Scale		0,0	-	10,0	7,0	6,3	±	2,6
mMRC Scale		0,0	-	4,0	3,0	2,5	±	1,2
GOLD Stage	I					5		3,1%
	II					46		28,8%
	III					74		46,3%
	IV					35		21,9%
Long term oxygen therapy						141		88,1%
NIMV						94		58,8%
Number of exacerbations in the previous year		0,0	-	12,0	2,0	3,1	±	3,0
Length of hospital stay (days)		2,0	-	25,0	7,0	7,4	±	2,8
Number of ICU admissions in the previous year		0,0	-	8,0	0,0	0,7	±	1,3

Data are presented as mean ± standard deviation. BMI: Body Mass Index , mMRC: Modified Medical Research Council , NIMV: Non-invasive Mechanical Ventilation , ICU: Intensive Care Unit

Supplementary Table S5B- Laboratory, PFT and echocardiographic values of the patients

	Min-Max			Median	Mean ±s.d./n-%		
HGB(g/dL)	7,8	-	19,4	14,0	14,0	±	2,2
HCT (%)	23,1	-	61,7	43,0	43,5	±	6,4
WBC (10^3/uL)	1,1	-	251,8	11,3	15,9	±	25,9
Neutrophil (10^3/uL)	0,0	-	34280,0	7880,0	9248,9	±	4975,6
PLT (10^3/uL)	2,2	-	69,5	23,1	25,7	±	10,3
Pct (%)	0,1	-	0,7	0,2	0,2	±	0,1
PDW	7,1	-	17,8	10,8	11,2	±	1,9
MPV (fL)	7,3	-	12,4	9,7	9,7	±	1,0
RDW-CV (%)	12,3	-	28,3	15,1	15,9	±	2,8
Eosinophil (10^3/uL)	0,0	-	1560,0	85,0	146,4	±	220,7
Eosinophil (%)	0,0	-	13,9	0,8	1,4	±	1,9
Lymphocyte (10^3/uL)	170,0	-	8280,0	1460,0	1599,7	±	900,5
NLR	0,6	-	100,8	5,6	7,9	±	9,6
PLR	5,1	-	1500,0	166,3	208,8	±	170,6
CRP (mg/L)	1,3	-	345,5	45	71,0	±	72,2
Procalcitonin (ng/mL)	0,0	-	11,2	0,45	0,7	±	1,1
Uric Acid (mg/dL)	1,1	-	11,4	5,6	5,7	±	1,9
Uric Acid / Creatinine	0,9	-	12,8	6,8	7,0	±	2,2
Total Protein (g/dL)	5,5	-	82,6	6,8	7,3	±	6,0
Albumin (g/dL)	0,4	-	39,0	3,6	3,8	±	2,9
pH	7,2	-	7,6	7,4	7,4	±	0,1
PCO2 (mmHg)	25,8	-	106,7	51,9	52,0	±	14,9
PO2 (mmHg)	28,6	-	367,0	55,2	66,6	±	39,5
O2 Saturation (%)	48,4	-	99,7	89,5	86,1	±	11,2
FEV1 (L)	0,4	-	25,2	1,0	1,2	±	2,0
FEV1 (%)	12,6	-	43,6	34,6	64,8	±	34,2
FVC (L)	0,5	-	48,4	1,7	2,2	±	4,7
FVC (%)	14,5	-	96,0	45,7	47,9	±	17,0
FEV1/FVC	26,6	-	94,9	56,3	58,8	±	13,2
EF (%)	22,0	-	60,0	60,0	55,4	±	7,8
PAP (mmHg)	20,0	-	83,0	30,0	35,4	±	13,1

Data are presented as mean ± standard deviation. HGB: Hemoglobin, HCT: Hematocrit, WBC: White Blood Cells, PLT: Platelet, Pct: Plateletcrit, PDW: Platelet Distribution Width, MPV: Mean platelet volüme, RDW-CV : RDW-CV : Erythrocyte distribution width - coefficient of variation, NLR: Neutrophil Lymphocyte Ratio, PLR :Platelet Lymphocyte Ratio, FEV1: Forced expiratory volume in first second , FVC: Forced vital capacity , EF: Ejection Fractio

n , PAP : Pulmonary Arterial Pressure

Supplementary Table S6. Logistic Regression Analysis for Procalcitonin

**Procalsitonin**	Univariate Model					Multivariate Model				
	OR	%95 confidence interval			p	OR	%95 confidence interval			p
Neutrophile	1,00	1,00	-	1,00	***0,008***	1,00	1,00	-	1,00	***0,008***
NLR	1,10	1,02	-	1,19	***0,011***					
CRP	1,01	1,00	-	1,01	***0,008***					

Supplementary Table S7. Logistic Regression Analysis for CRP

**CRP**	Univariate Model					Multivariate Model				
	OR	%95 confidence interval			p	OR	%95 confidence interval			p
HCT	0,92	0,88	-	0,98	***0,004***	0,918	0,867	-	0,972	***0,003***
Eosinophile	1,00	0,99	-	1,00	***0,009***	0,997	0,995	-	0,999	***0,014***
Eosinophile	0,68	0,53	-	0,88	***0,003***					
Lymphocyte	1,00	1,00	-	1,00	***0,011***					
Neutrophile	1,00	1,00	-	1,00	***0,000***	1,00	1,00	-	1,00	***0,001***
NLR	1,19	1,08	-	1,31	***0,000***					
PLR	1,00	1,00	-	1,01	***0,015***					

Procalsitonin	1,92	1,10	-	3,36	***0,022***					
pH	289,9	2,3	-	36518,0	***0,022***					
PCO2	0,98	0,96	-	1,00	***0,033***					
PO2	0,99	0,98	-	1,00	***0,047***					

Supplementary Table S8. Logistic Regression Analysis for NLR

NLR	Univariate Model					Multivariate Model				
	OR	%95 confidence interval			p	OR	%95 confidence interval			p
Eosinophile	1,00	0,99	-	1,00	***0,003***					
Eosinophile	0,56	0,41	-	0,75	***0,000***	0,610	0,450	-	0,825	***0,001***
CRP	1,01	1,00	-	1,01	***0,001***	1,007	1,001	-	1,012	***0,016***
Procalsitonin	2,34	1,25	-	4,37	***0,008***					
